# Variación longitudinal comparativa de los anticuerpos totales, IgG e IgA contra el SARS-CoV-2 en receptores de la vacuna BNT162b2

**DOI:** 10.1515/almed-2021-0095

**Published:** 2022-01-05

**Authors:** Giuseppe Lippi, Gian Luca Salvagno, Brandon M. Henry, Laura Pighi, Simone De Nitto, Gianluca Gianfilippi

**Affiliations:** Sección de Bioquímica Clínica, Universidad de Verona, Verona, Italia; Servicio de medicina de laboratorio, Hospital Pederzoli, Peschiera del Garda, Italia; Laboratorio clínico, Sección de Nefrología e Hipertensión, Cincinnati Children’s Hospital Medical Center, Cincinnati, OH, USA; Disease Intervention & Prevention and Population Health Programs, Texas Biomedical Research Institute, San Antonio, TX, USA; Dirección médica, Pederzoli Hospital, Peschiera del Garda, Italia

**Keywords:** anticuerpos, COVID-19, respuesta inmune, SARS-CoV-2, vacuna

## Abstract

**Objetivos:**

El objeto del presente estudio es resumir la variación de una amplia serie de anticuerpos contra el SARS-CoV-2 en sujetos receptores de la vacuna de mARN BNT162b2, en un periodo de seis meses.

**Métodos:**

La población del estudio estaba formada por 84 profesionales sanitarios seronegativos al SARS-CoV-2 en situación basal (media de edad: 45 años, 53.6% mujeres), que recibieron la vacuna de mARN denominada BNT162b2. Se tomó una muestra de sangre previamente a la primera y segunda dosis de la vacuna, así como al cabo de 1, 3 y 6 meses. Se determinó el título sérico de los siguientes anticuerpos contra el SARS-CoV-2: anticuerpos totales específicos para el dominio RBD (dominio de unión al receptor), IgG contra la proteína trimérica espicular *(Spike),* IgG específica para el dominio RBD, e IgA contra el receptor S1 de la proteína trimérica espicular del SARS-CoV-2.

**Resultados:**

Todos los anticuerpos alcanzaron su nivel máximo al mes de recibir la vacunación, pero disminuyeron de forma significativa posteriormente. La tasa media de decrecimiento a los 6 meses fue de −95% para IgG anti- RBD, −85% para IgG contra la proteína trimérica espicular, −73% para IgA anti-S1 y −56% para los anticuerpos totales anti-RBD del SARS-CoV-2, respectivamente. El tiempo medio para la seronegatividad fue de 579 días para los anticuerpos totales anti-RBD SARS-CoV-2, 271 días para IgG contra la proteína trimérica espicular, 264 días para IgG anti-RBD, y 208 días para IgA anti-S1 SARS-CoV-2, respectivamente. A los seis meses, la tasa de sujetos seropositivos se había reducido del 98–100% en el momento de su nivel máximo al 50–100%. La variación entre individuos, en términos de reducción de anticuerpos contra el SARS-CoV-2, a los seis meses fue del 3–44% con respecto al nivel máximo.

**Conclusiones:**

Los resultados de este estudio serológico demuestran que el título de anticuerpos contra el SARS-CoV-2 disminuyó a los seis meses de haber recibido la vacuna BNT162b2, con un periodo medio hasta alcanzar la seronegatividad a los anticuerpos IgG/IgA de 7–9 meses, lo que evidencia la necesidad de administrar dosis de refuerzo a los seis meses, aproximadamente, de la última dosis.

## Introducción

La pandemia actual de síndrome respiratorio agudo severo por coronavirus 2 (SARS-CoV-2) ha planteado desafíos sin precedentes a la salud humana, la sociedad y la economía. Con más de cinco millones de muertes a fecha de noviembre de 2021, la enfermedad de coronavirus 2019 (COVID-19) ya puede ser considerada la mayor catástrofe sufrida por la Humanidad, desde la pandemia de gripe española de 1918–1919 [1]. A pesar de la recomendación e incluso imposición en algunos países del uso generalizado de medidas físicas de prevención, como la distancia social o el confinamiento, el uso de mascarillas y la higiene de manos, la vacunación frente al COVID-19 parece ser la única forma tangible de prevenir o limitar la expansión del virus [2]. Aunque se han desarrollado algunas vacunas contra la COVID-19 (inactivadas, basadas en proteínas, ADN o ARN), y su uso ha sido aprobado por las agencias sanitarias de todo el mundo en adultos e incluso niños [3], existen considerables diferencias en su eficacia. Concretamente, un metaanálisis de estudios de evidencia del mundo real apunta que las vacunas de mARN (p.ej. BNT162b2 y mRNA-1273) son las que mayor eficacia muestran, frente a las vacunas de adenovirus (p.ej., ChAdOx1 y Ad26.COV2.S) e inactivadas (p.ej., CoronaVac) (eficacia del 85–100% frente al 65–91% en la prevención de la COVID-19 sintomática) [4]. Pese a la eficacia notable de estas vacunas contra la COVID-19, que es considerablemente superior a la de la vacuna contra la gripe a la hora de prevenir una infección sintomática [5], existen evidencias claras de que la protección lograda disminuye con el tiempo, concretamente, a los seis meses de la vacunación. Estos hallazgos sustentan la necesidad de administrar una dosis de refuerzo de la vacuna contra la COVID-19 a una gran parte de la población [6]. Dado que la eficacia de la vacuna contra la COVID-19 depende en gran medida de la presencia y persistencia de anticuerpos neutralizadores del SARS-CoV-2 [7], el objeto del presente estudio es determinar la variación de una amplia variedad de anticuerpos contra el SARS-CoV-2, en sujetos receptores de la vacuna de mARN BNT162b2, en un periodo de seis meses.

## Materiales y métodos

Inicialmente, nuestra población de estudio estaba formada por 100 profesionales sanitarios seronegativos al SARS-CoV-2, del Hospital de Peschiera del Garda (Italia), que recibieron la vacuna de mARN de Pfizer/BioNTech BNT162b2 (Comirnaty, Pfizer Inc., Nueva York, EE.UU) en dos dosis de 30 µg, espaciadas en 21 días). Se tomaron muestras de sangre inmediatamente antes de administrar la primera y segunda dosis de la vacuna, y a los 1, 3 y 6 meses de la segunda dosis. El protocolo de este estudio ha sido descrito detalladamente en artículos anteriores [8], [9], [10]. Se separó el suero mediante centrifugación a 3,000×*g* durante 15 minutos, a temperatura ambiente. Se determinaron los títulos séricos de los siguientes anticuerpos contra el SARS-CoV-2: anticuerpos anti-RBD (Roche Elecsys Anti-SARS-CoV-2 S inmunoensayo quimioluminiscente en Roche Cobas 6000; Roche Diagnostics, Basel, Suiza; resultado positivo: ≥0.82 BAU/mL); IgG contra la proteína trimérica espicular *Spike* (IgG anti-espicular trimérico con la prueba DiaSorin en Liaison XL; DiaSorin, Saluggia, Italia; ≥33.8 BAU/mL); IgG anti-RBD (ACCESS SARS-COV-2 IgG II en ACCESS 2; Beckman Coulter Inc., Brea, CA, EE.UU; resultado positivo: ≥10 AU/mL); e IgA contra el receptor S1 de la proteína espicular del SARS-CoV-2 (Anti-SARS-CoV-2 ELISA IgA; Euroimmun, Lübeck, Alemania; resultado positivo: ≥1.1 AU/mL). Todas estas pruebas mostraron una excelente correlación con las pruebas de neutralización de referencia [11], [12], [13]. Los resultados de las pruebas se expresaron en la unidad de medida indicada por el fabricante (unidad de anticuerpos de unión [BAU], cuando se disponía de ella), y como el coeficiente de variación entre el valor obtenido y el valor basal (esto es, [valor en un punto temporal]/[valor basal]. Los resultados se expresaron como mediana y rango intercuartílico (RIC). Todos los participantes firmaron un consentimiento informado por escrito antes de recibir la vacuna y participar en el estudio serológico longitudinal. El estudio se realizó de conformidad con la Declaración de Helsinki y fue aprobado por el Comité Ético de las provincias de Verona y Rovigo (59COVIDCESC; 3 de noviembre de 2021).

## Resultados

**Figura 1:**
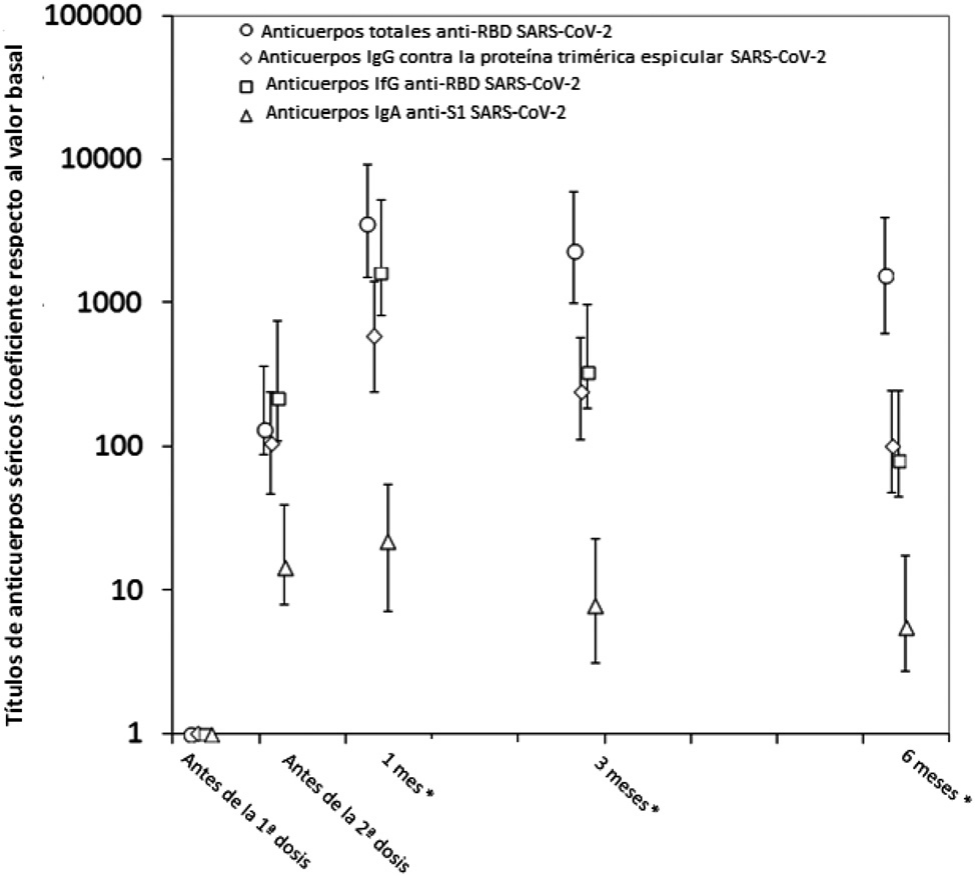
Cinética de los anticuerpos séricos totales anti-RBD (dominio de union al receptor), IgG contra la proteína trimérica espicular, IgG anti-RBD, e IgA anti-S1 de la proteína espicular, en sujetos seronegativos receptores de la vacuna de mARN BNT162b2. *Tras la segunda dosis de la vacuna.

**Figura 2:**
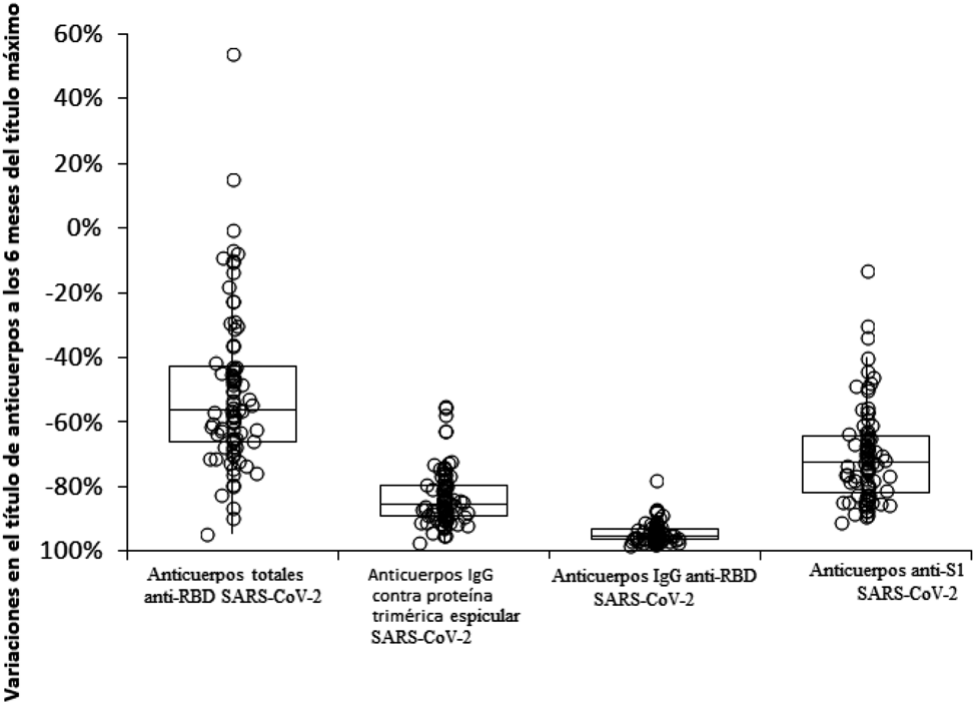
Reducción porcentual seis meses después de que alcancen el nivel máximo los anticuerpos séricos totales anti-RBD (dominio de union al receptor), IgG contra la proteína trimérica espicular, IgG anti-RBD, e IgA anti-S1 de la proteína espicular, en sujetos seronegativos receptores de la vacuna de mARN BNT162b2.

La muestra final estaba compuesta por 84 profesionales sanitarios seronegativos al SARS-CoV-2 (edad media 45 años, RIC 31–35 años; 53.6% mujeres), habiendo perdido a 16 sujetos durante el seguimiento. En la Figura 1 se muestra la variación en los títulos de diferentes anticuerpos a lo largo de seis meses, en un estudio serológico longitudinal. Todos los anticuerpos alcanzaron sus niveles máximos un mes después de la vacunación, siendo los anticuerpos anti-RBD SARS-CoV-2 los que mostraron un mayor coeficiente de variación con respecto al valor basal (mediana de coeficiente, 3,550; RIC, 2,040–5,682), seguido de los anticuerpos IgG anti-RBD (mediana del coeficiente, 1,620; RIC, 795–3,618), anticuerpos IgG contra la proteína trimérica espicular (mediana del coeficiente, 584; RIC, 342–834) y los anticuerpos IgA anti-S1 SARS-CoV-2 (mediana de coeficiente, 22; RIC, 15–32). Posteriormente, se observó una disminución notable de los títulos de anticuerpos, con las mediana de la tasa de reducción a los seis meses del cociente entre el valor actual de anticuerpos y el nivel máximo de anticuerpos (esto es, [valor a los seis meses]/[valor basal]) disminuyendo un −95% (RIC, entre −93% y −96%) en los anticuerpos IgG anti-RBD, −85% (RIC, entre −80% y −89%) en los anticuerpos IgG contra la proteína trimérica espicular, −73% (RIC, entre −64% y −81%) en los anticuerpos IgA anti-S1, y −56% (RIC, entre −43% y −66%) en los anticuerpos totales anti-RBD SARS-CoV-2 (Figura 2). Mediante ajuste logarítmico, calculamos que la mediana de tiempo hasta volver al estado de seronegatividad sería de 579 días para los anticuerpos totales anti-RBD, 271 días para la IgG contra la proteína trimérica espicular, 264 días para la IgG anti-RBD, y 208 días para los anticuerpos IgA anti-S1 del SARS-CoV-2, respectivamente.

La tasa de sujetos seropositivos a lo largo del estudio se muestra en la Figura 4, habiéndose incrementado del 0% al 73–96% tras la primera dosis y al 98–100% tras la segunda dosis de la vacuna BNT162b2. Por otro lado, la tasa de seropositivos disminuyó al 70–100% a los tres meses de la segunda dosis, para descender finalmente al 50–100% a los seis meses de la segunda dosis de la vacuna. Concretamente, los IgA anti-S1 SARS-CoV-2 fueron los que mostraron la mayor seronegativización (50%), mientras que todos los sujetos seguían siendo positivos en anticuerpos anti-RBD del SARS-CoV-2 a los seis meses. El coeficiente de variación en la reducción con respecto al nivel máximo registrado, a los seis meses de la segunda dosis, fue del 3% en los anticuerpos IgG anti-RBD, el 10% en los anticuerpos IgG contra la proteína trimérica espicular, el 20% para los anticuerpos IgA anti-S1 del SARS-CoV-2, alcanzando hasta el 44% en los anticuerpos anti-RBD, respectivamente.

**Figura 3:**
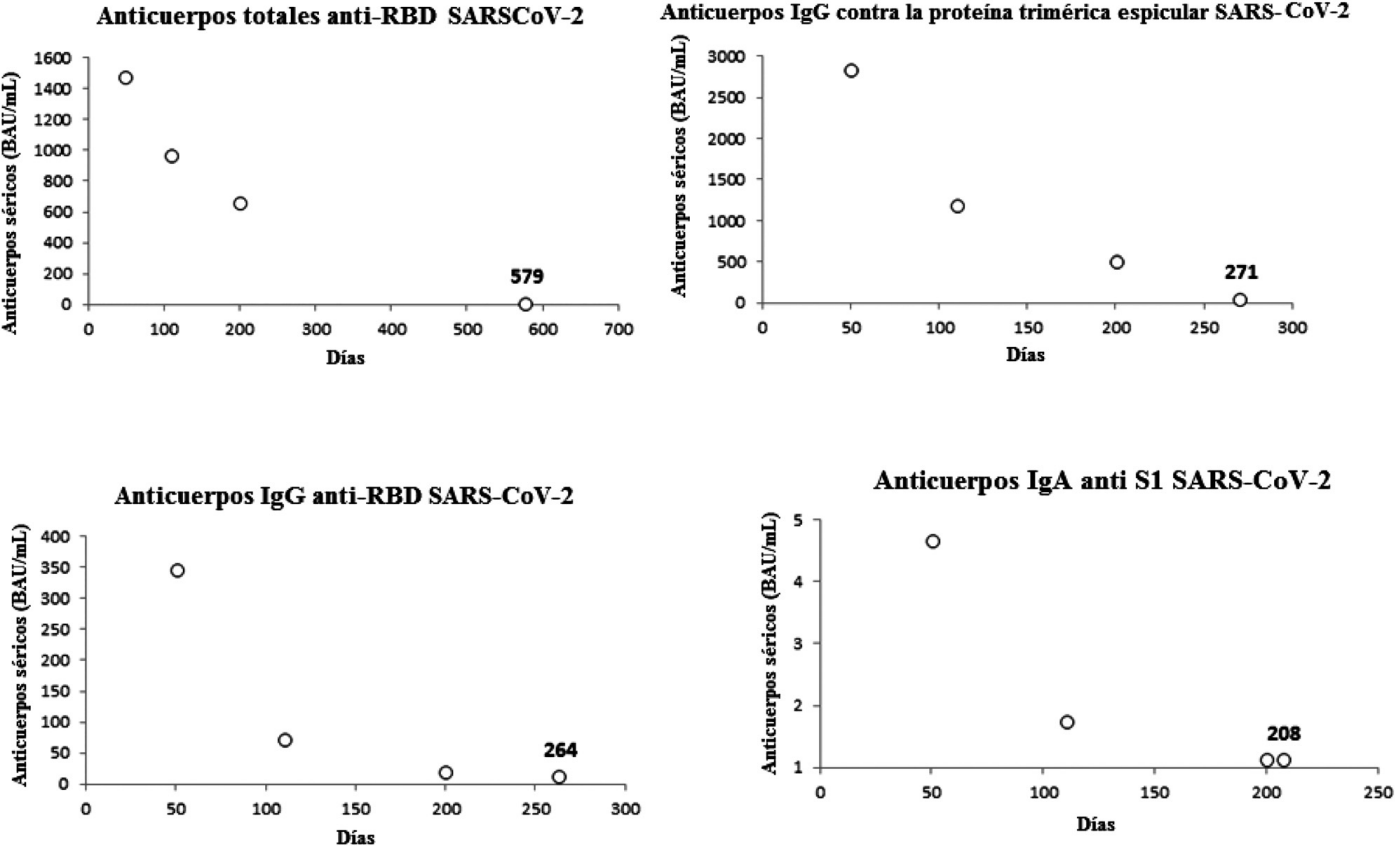
Tiempo medio estimado de seronegativización después de que alcancen el nivel máximo los anticuerpos séricos totales anti-RBD (dominio de union al receptor), IgG contra la proteína trimérica espicular, IgG anti-RBD, e IgA anti-S1 de la proteína espicular, en sujetos seronegativos receptores de la vacuna de mARN BNT162b2.

**Figura 4:**
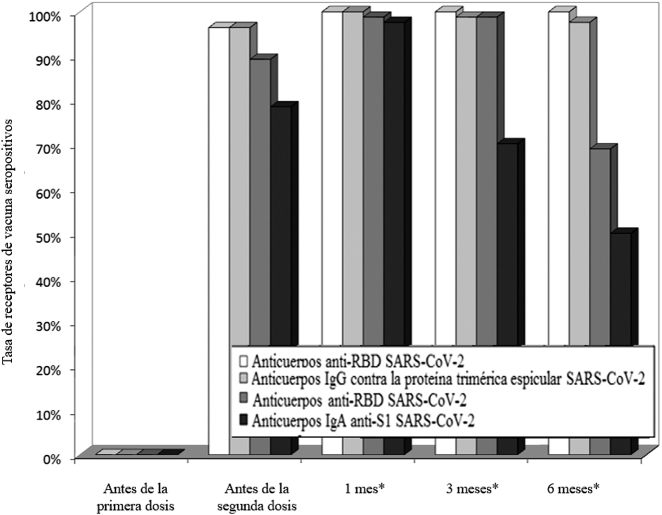
Tasa de sujetos receptores de la vacuna de mARN BNT162b2 seronegativos a los anticuerpos séricos totales anti-RBD (dominio de union al receptor), IgG contra la proteína trimérica espicular, IgG anti-RBD, e IgA anti-S1 de la proteína espicular. *Tras la segunda dosis de la vacuna.

## Discusión

Los resultados obtenidos en el presente estudio serológico longitudinal demuestran que la mediana del título de una amplia variedad de anticuerpos contra el SARS-CoV-2 disminuyó significativamente tras la administración de la segunda dosis de la vacuna BNT162b2, con la mitad de los receptores volviendo a ser seronegativos a los anticuerpos IgA anti-S1 a los seis meses. Un número significativo de los sujetos también acabaron siendo seronegativos a los anticuerpos IgG anti-RBD (∼30%), mientras que la mayoría (>98%) mantuvo los niveles de anticuerpos IgG contra la proteína trimérica espicular y anticuerpos totales anti-RBD SARS-CoV-2 por encima de sus respectivos umbrales de positividad.

Las variaciones longitudinales en los anticuerpos contra el SARS-CoV-2 observadas a lo largo de 6 meses, tras completar la pauta de vacunación de BNT162b2 en nuestra población, suscita algunas reflexiones en términos biológicos y clínicos. Los estudios recientes sobre la eficacia de las vacunas de mARN contra el COVID-19 (p.ej. BNT162b2 y mARN-1273) arrojan datos preocupantes sobre la protección conferida contra todo tipo de infección por SARS-CoV-2, disminuyendo los valores de protección del 87–89% al 43–58% a los seis meses de la vacunación [14]. Por otro lado, durante el mismo periodo, dichas vacunas sí mostraron una protección estable frente a la hospitalización (del 88–93% al 77–92%) [15]. La eficacia de la vacuna parece depender de los títulos de anticuerpos neutralizantes contra el SARS-CoV-2 [7]. Esto parece quedar demostrado por el hecho de que la mayor reducción y seronegatividad observadas en nuestro estudio correspondan a los anticuerpos IgA anti-S1, lo que explicaría que la protección frente al riesgo de infección por SARS-CoV-2, tanto sintomática como asintomática, también se reduzca con el tiempo, según los últimos estudios epidemiológicos. Esta relación es lógica, ya que este tipo de anticuerpos constituye la primera línea de defensa de la mucosa frente a multitud de enfermedades infecciosas (especialmente respiratorias) [16, 17]. Por otro lado, el relativamente mejor mantenimiento de la inmunidad frente a la infección sintomática y aguda por SARS-CoV-2 a lo largo del tiempo parece explicarse por el hecho de que también se mantengan en el tiempo los títulos de anticuerpos anti-RBD totales y anticuerpos IgG contra la proteína trimérica espicular, tal como se muestra en la Figura 3. Este aspecto, unido al desarrollo y persistencia de la memoria inmune y de la inmunidad mediada por células tras la vacunación contra la COVID-19, pueden ofrecer protección frente a la expansión local (nasofaríngea, pulmonar) y sistémica del virus [18, 19]. No obstante, no resulta tranquilizador que los niveles séricos de estos dos tipos de anticuerpos hayan mostrado una reducción gradual considerable tras alcanzar sus niveles máximos (−56% y −73%, respectivamente), lo que indica que los niveles de estas inmunoglobulinas también podrían disminuir hasta quedar por debajo del umbral de positividad en un periodo relativamente corto de tiempo, que nosotros estimamos en entre 9 y 19 meses.

La mayor variación entre individuos en la tasa de disminución de anticuerpos IgA anti-S1 y anti-RBD del SARS-CoV-2 observada en nuestra población (20% y 445, respectivamente) es otro aspecto importante, lo que subraya la importancia de realizar una monitorización serológica tras la vacunación [20]. Esta práctica permitiría identificar anticipadamente a aquellos sujetos que experimenten una mayor disminución de los niveles séricos de anticuerpos [21], a los que se debe dar prioridad en la administración de la dosis de refuerzo [22]. Los datos obtenidos sobre el tiempo hasta la seronegativización coinciden con las últimas recomendaciones del Comité Asesor sobre Prácticas de Vacunación, en relación a la administración de dosis de refuerzo de las vacunas contra la COVID-19 [22]. De hecho, a excepción de los anticuerpos totales anti-RBD, cuya seronegativización probablemente se produzca a los 19 meses, el tiempo medio de seronegativización a otros tipos de anticuerpos en nuestro estudio variaba entre los 7 y los 9 meses (Figura 3), lo que refuerza la recomendación de administrar dosis de refuerzo adicionales de la vacuna contra la COVID-19 tras la última dosis de la vacuna, especialmente en los grupos de población más vulnerables.
